# Revisiting the Vertical Osteotomy Technique for the Removal of a 20-Year-Old Tibial Intramedullary V-Nail: A Case Report

**DOI:** 10.7759/cureus.63653

**Published:** 2024-07-02

**Authors:** Ashutosh Lohiya, Nareshkumar Dhaniwala, Saksham Goyal, Hardik Patel

**Affiliations:** 1 Department of Orthopedics, Jawaharlal Nehru Medical College, Datta Meghe Institute of Higher Education and Research, Wardha, IND

**Keywords:** difficulty in removal, vertical osteotomy, implant removal, tibial fracture, intramedullary nail

## Abstract

Vertical osteotomy is a procedure occasionally used for the removal of intramedullary nails when the nail has become jammed, either due to expansion after initial fixation or the formation of a new bone around the nail. Implant removal of any type is usually performed when it is either recommended by the doctor or in response to the complaints of the patients, like sensations of pain, disorder, and infection associated with the potential complications of the given implant. There are different types of cases, which range from a simple procedure of K-wire removal or more complex procedures like intramedullary nail or plate removal. During the removal of implants, certain unforeseen complications can occur such as excessive bleeding, neurovascular deficit, and some other issues related to implants like breakage of screws or implants while removing it, which might lead to its inability to be removed. We present here a technique of vertical osteotomy that was used for the removal of implants in cases of long-term implant retention, which leads to difficulty in removing it.

## Introduction

Implant removal is one of the most common orthopedic procedures in the West which was used to fix a broken bone [1]. Tibial shaft fractures are mostly managed by intramedullary nailing which remains one of the most popular forms of treatment. The general trend shows that intramedullary nailing is used in approximately 70-80% of tibial shaft fractures in developed countries where the technique and resources are readily available It has many benefits when compared to other methods that are used for fixing the fracture such as soft tissue dissection is minimal, fracture alignment is better and early full weight bearing mobilization [2]. However, sometimes it can turn into a more serious complication when the nail has been entered from an unusual place and is tried to be removed after a very long time. There is still a question of whether or not implants should be removed after fracture healing. Removal of implants associated with problems would be hard to dispute [3].

We here present a case of a 48-year-old male patient who underwent closed reduction and internal fixation with intramedullary V-nail surgery for a distal 1/3rd tibial shaft fracture 20 years ago. The patient came to the hospital with a request for removal of the implant since he was having a complaint of discharging sinus from the entry point along with pain as he was worried about the implant's long-term retention.

The V-nail removal was tried using conventional methods like nail removal by bone hooks in this case but due to its failure, it was decided that a classic vertical osteotomy technique could be used to execute the extraction process. This technique helped in loosening the well-integrated nail and facilitating its removal [4].

This case report helps to draw attention to the vertical osteotomy technique that was used for removing the difficult and complicated intramedullary nail, especially when the nail was positioned at the unusual site or had been in place for a long time. 

The standard method of removal of old-type intramedullary nails (open/slotted/v-shaped) includes engaging a bone hook into the eye of the nail and back hammering it for removal. The newer types of nails are circular and closed and have a threaded proximal end. For its removal, the corresponding threaded bolt is to be engaged into the proximal end and back hammering is done over the extractor. It also emphasizes how crucial it is to be up to date with old methods because they can offer workable answers in challenging clinical situations.

## Case presentation

**Figure 1:**
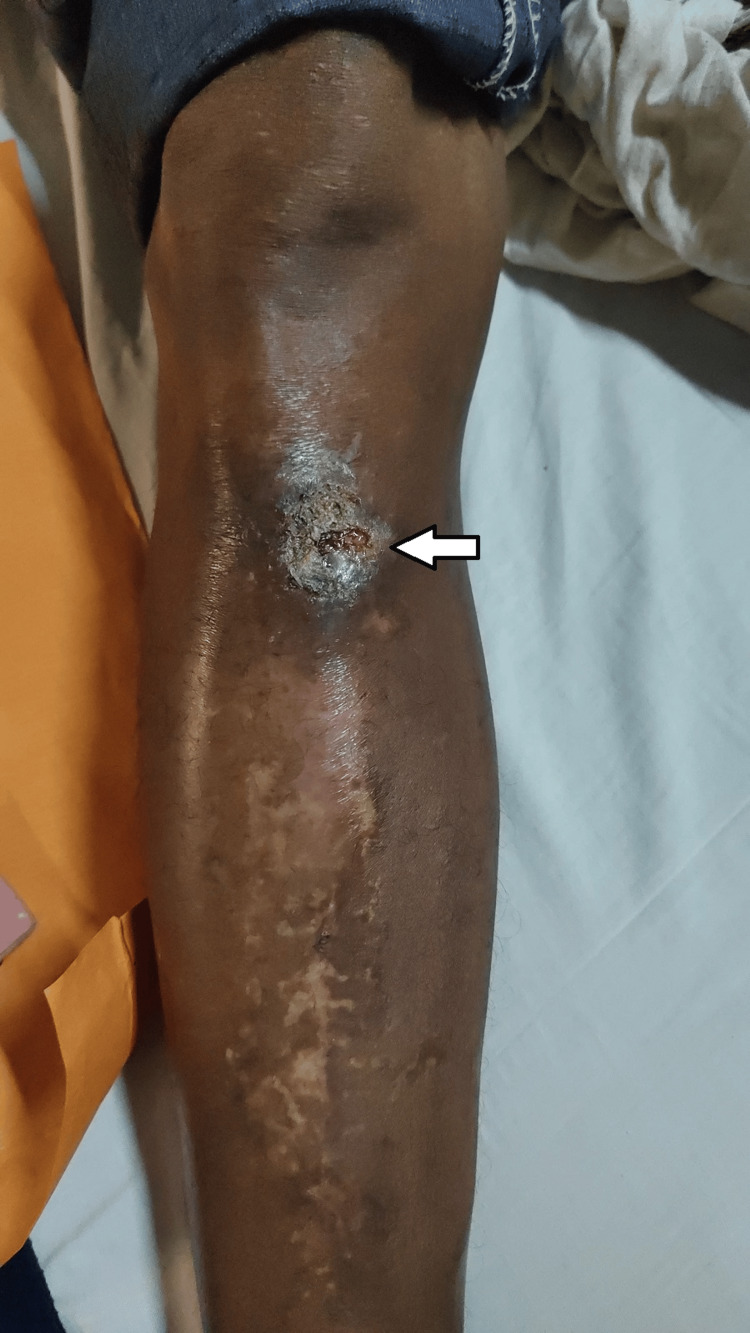
Clinical image showing discharging sinus from the site of entry of the V-nail

A 48-year-old male patient came to the orthopedic OPD (Outpatient Department) with a complaint of discharging sinus from the site of V-nail entry which was atypical in position at proximal 1/3rd shaft tibia right side for three months. The patient was operated on 20 years back for a distal 1/3rd shaft tibia fracture on the right side with closed reduction and intramedullary V-nail insertion from an unusual site at a civil hospital. The patient was able to bear weight and walk after surgery. The patient was asymptomatic for the past 19 years and then started complaining of discharge from the entry point which was minimal at onset and progressively increasing and used to require dressing on a daily basis. The patient then also complained about slight pain while walking. Because of the continuous discharge many years after the initial surgery from the site and pain in the limb with concerns regarding the retained intramedullary nail in his right tibia, the patient requested elective implant removal (Figure 1).

On examining the patient, the right lower limb appeared well-aligned, with no obvious deformities. However, there was discharging sinus which was noted many years after the initial surgery due to the nail end irritating the overlying soft tissues, located at the antero-medial aspect of the proximal tibia, corresponding to an unusual entry point of the intramedullary V-nail in this case. Range of movements at the knee and ankle joints were within normal limits, and neurovascular status was intact.

**Figure 2:**
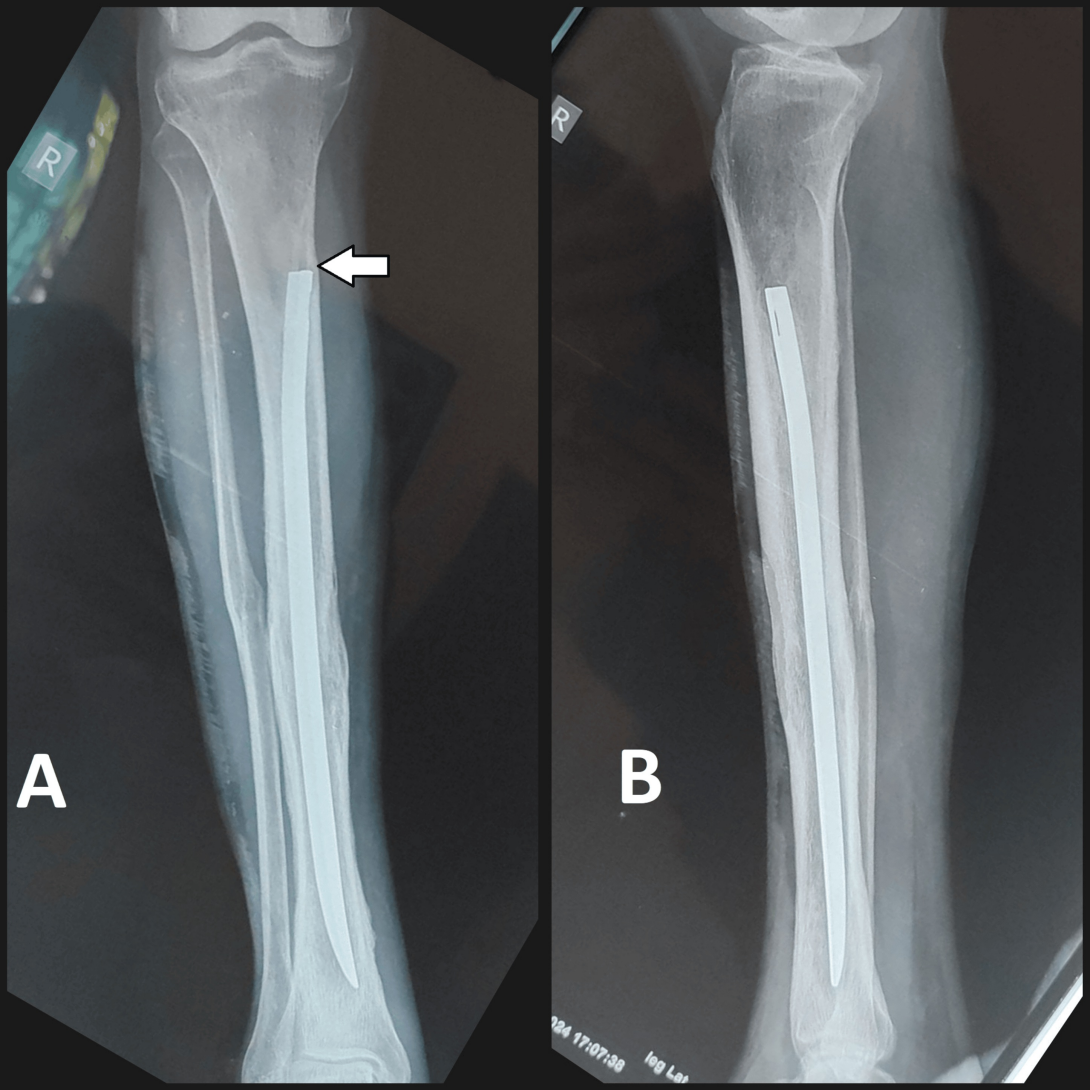
Pre-operative radiograph: (A) Antero-posterior view and (B) lateral view with V-nail in situ (white arrow showing the entry point of the V-nail)

Radiographic evaluation in the form of X-rays revealed a well-united tibial shaft fracture with the V-nail in situ. Notably, the nail appeared to be in an unusual position, with its proximal end lying closer to the anterior cortex of the tibia on the proximal 1/3rd, rather than the typical central placement within the medullary canal (Figure 2).

After a thorough explanation of the potential risks and benefits, the patient decided to proceed with the procedure and gave the consent for removal of the V-nail. Considering the atypical positioning of the nail and the long duration, the removal process was planned.

Intra-operatively, standard techniques for nail removal, including the use of specialized extraction instruments like bone hooks, were initially attempted. However, these efforts of removal proved to be unsuccessful due to the well-integrated nature of the nail within the tibial canal.

**Figure 3:**
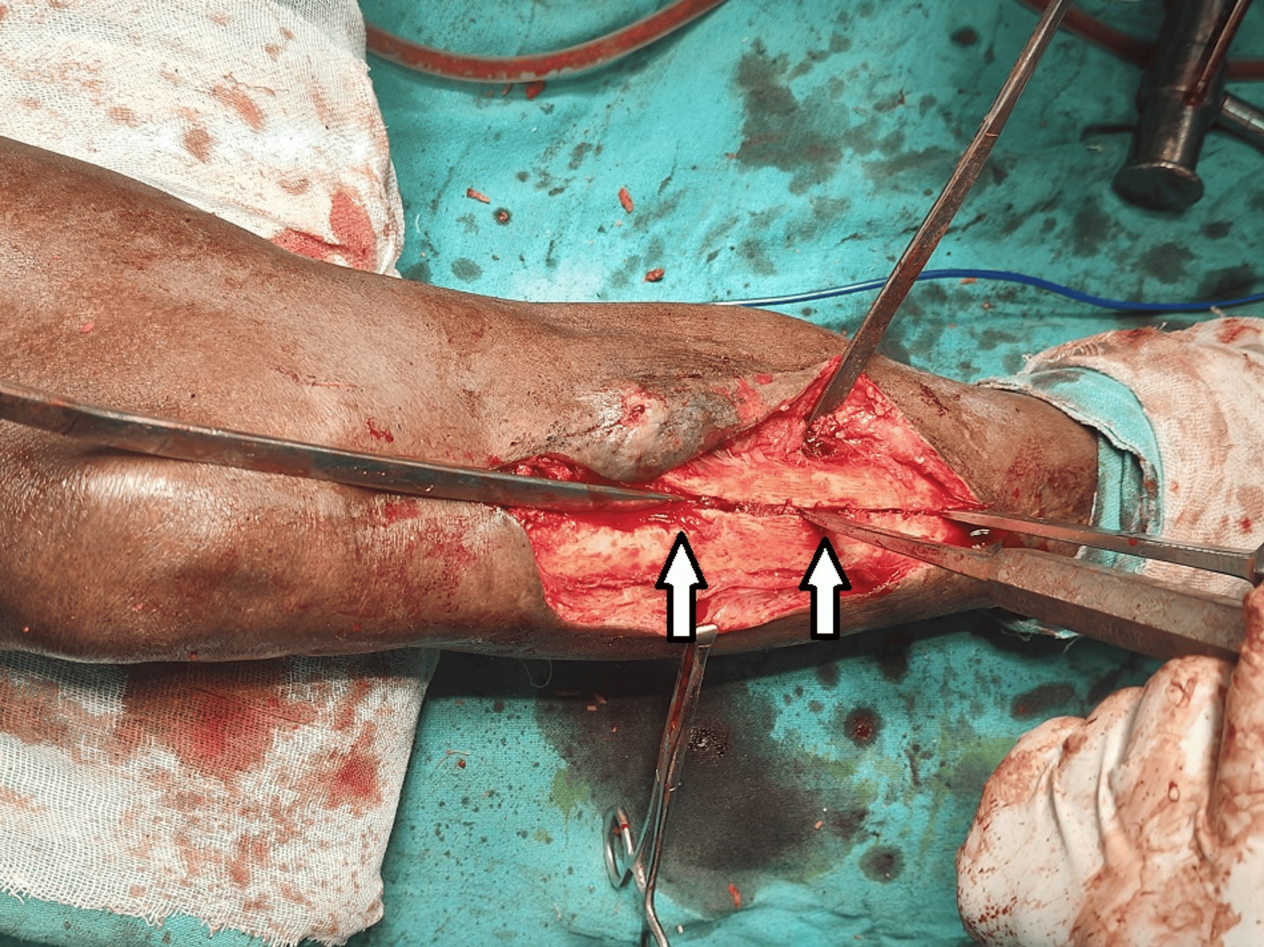
Intra-operative image showing vertical osteotomy being done to loosen the V-nail

As the removal of the nail was difficult and challenging, an old technique of vertical osteotomy approach was applied to facilitate the nail's removal. A 10-cm longitudinal osteotomy along the line of nail was performed on the antero-medial aspect of the tibial shaft, starting from the entry point of the nail distally. This osteotomy helped in creating a vertical bony split, which provided a space for osteotome to pass and loosen the well-integrated nail in the intramedullary canal (Figure 3).

**Figure 4:**
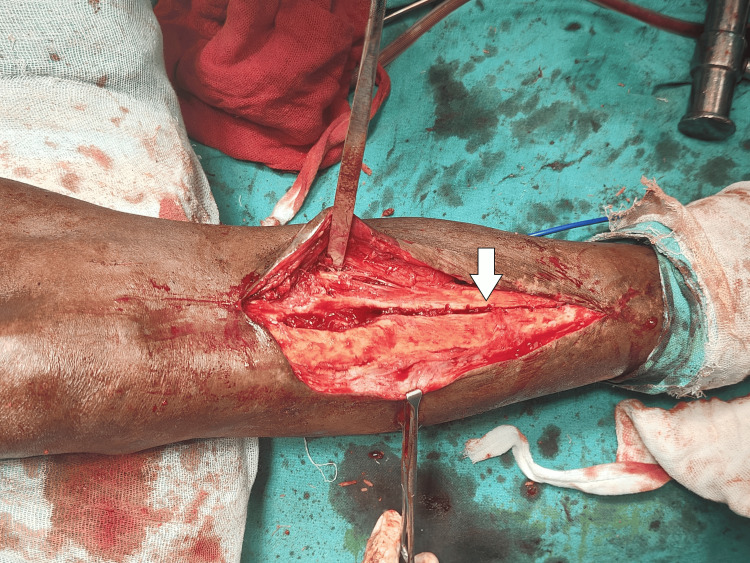
Intra-operative image showing vertical osteotomy following V-nail removal

Through the vertical osteotomy, the nail's position could be clearly visualized, and specialized instruments were used to disengage it from the surrounding bone. With careful manipulation, the nail was gradually loosened and successfully extracted from the tibial canal (Figure 4).

**Figure 5:**
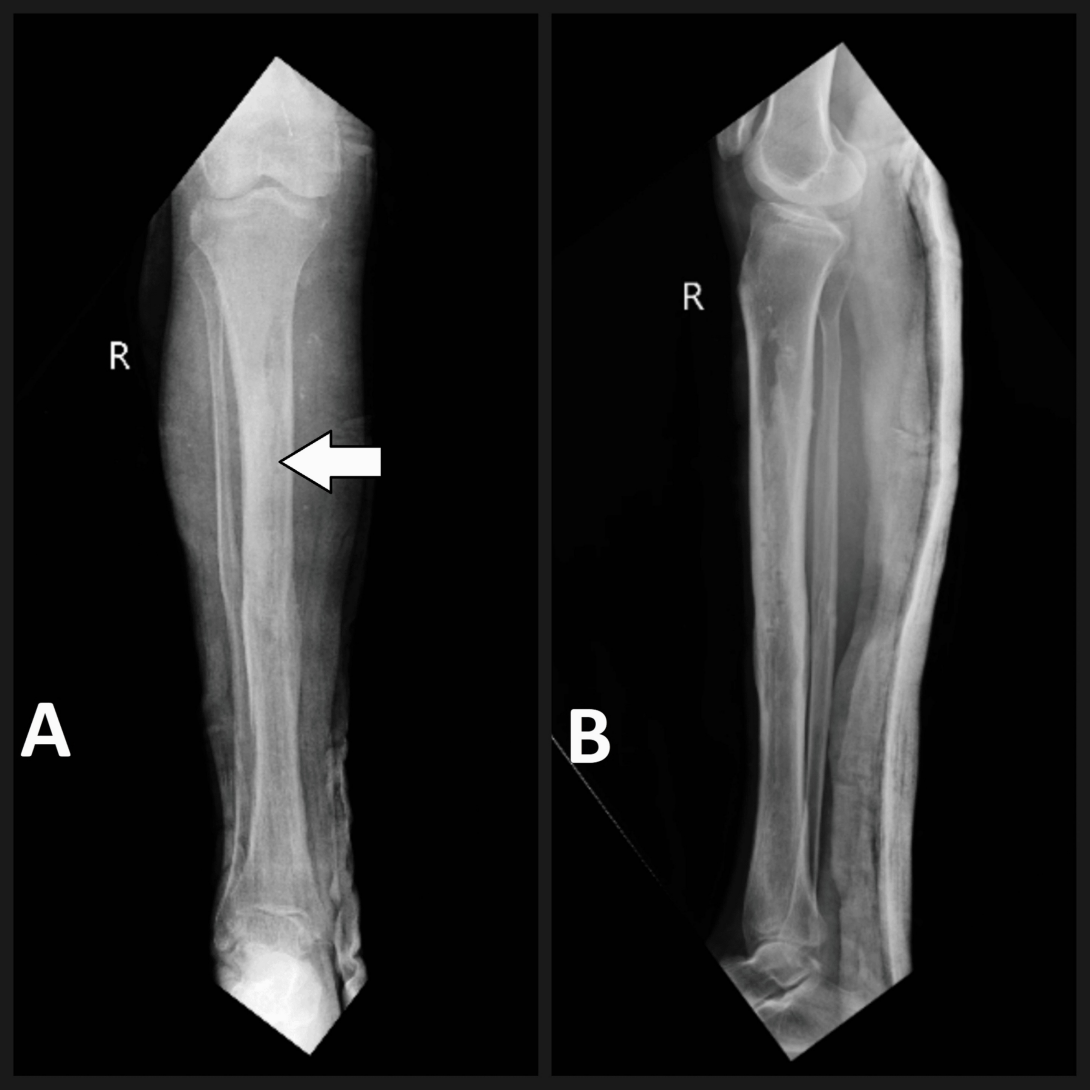
Post-operative radiograph: (A) Antero-posterior view (white arrow showing the line of vertical osteotomy) and (B) lateral view

Thorough washing and excision of the sinus tract were done, suturing was done in layers and the limb was stabilized using the above knee slab for initial days. The patient was advised for non-weight bearing for 4 to 6 weeks. Post-operative X-rays were done and found to be satisfactory (Figure 5).

## Discussion

The removal of long-standing implants like intramedullary nails, particularly those in unusual positions, can present significant challenges to orthopedic surgeons. While numerous case reports have documented successful implant removal using standard techniques, a subset of cases require more unconventional approaches, as illustrated in our case report.

Karladani et al. conducted a retrospective review in which they operated 10 cases where the intramedullary nail removal had proved to be challenging. They stressed the different approaches that were used in these cases, such as the use of specialized extraction instruments, flexible reamers, and, in certain cases, window osteotomies [5]. They emphasized the importance of preoperative planning and the need to adapt to intraoperative complications. They reported successful nail removal in all cases.

Arif et al. reported the removal of a well-integrated Intramedullary nail in a patient who was managed 15 years ago for a tibial fracture with the use of a vertical osteotomy procedure [6]. They also emphasized the advantages of this method, such as the ability to disengage the nail directly and handle it more skillfully, which results in a successful extraction of the nail.

Pan and Reuben reported a case where femoral window osteotomy was done in a case to remove a distal femoral nail that had been in place for more than 20 years [7]. He and Li described a case where a long-standing intramedullary nail was removed with the use of a tibial window osteotomy, emphasizing the value of this approach in cases where standard techniques fail and lead to complications [8].

Our experience, in this case, is consistent with numerous other studies, emphasizing the significance of other alternative procedures, like window methods or osteotomies, when dealing with challenging cases of intramedullary nail removals [9]. Even though these procedures might be more invasive, they provide direct access to and visualization of the implant, increasing the chances of that implant being removed successfully and avoiding any iatrogenic complication.

The standard method of removal of old-type intramedullary nails (open/slotted/V-shaped) includes engaging a bone hook into the eye of the nail and back hammering it for removal. the newer types of nails are circular and closed and have threaded proximal ends. For its removal, the corresponding threaded bolt is to be engaged into the proximal end and back hammering is done over the extractor. On failure of the above attempts in the older type of nail or the new type of nail, vertical osteotomy of the shaft of the bone comes to the rescue as the intramedullary canal can be partially opened by this method, and nail removal becomes possible. this includes doing an osteotomy commonly starting from the proximal part of the nail along the shaft downwards. The osteotomy is marked and the cortex is weakened by drilling multiple drill holes along the line. Osteotomy is completed with the help of an osteotome and hammer along the line of drill holes and the osteotomy line is opened to widen the medullary cavity, thus making the canal wider and nail removal easier. However, knowing these old techniques and the experience of surgeons can be quite helpful when handling difficult and complicated situations [10].

## Conclusions

In conclusion, our case report helps in contributing to research that emphasises the possible challenges that can be involved in intramedullary nail removal, especially when it comes to implants that are at unusual position or have been in place for a long duration. Surgeons should always try normal techniques first, but when faced with difficult situations, they should be ready to adjust according to situation and think out of the box for other options, including osteotomies or window techniques. Knowing about the old methods can be helpful in cases with complications and difficulties to improve the patient outcomes in the long run.
